# Paragangliomas of the head and neck: a contemporary review

**DOI:** 10.1530/EO-22-0080

**Published:** 2022-11-10

**Authors:** Nathan J Graham, Joshua D Smith, Tobias Else, Gregory J Basura

**Affiliations:** 1 Department of Otolaryngology – Head & Neck Surgery, University of Michigan Medical School, Ann Arbor, Michigan, USA; 2 Department of Internal Medicine, Division of Metabolism, Endocrinology, and Diabetes, University of Michigan Medical School, Ann Arbor, Michigan, USA

**Keywords:** paraganglioma, head and neck, succinate dehydrogenase, carotid body, jugular

## Abstract

Head and neck paragangliomas (HNPGLs) are slow-growing, vascular, typically benign tumors whose growth may induce significant lower cranial nerve deficits. While most tumors arise sporadically, a significant portion is associated with defined genetic syndromes. While surgical resection has historically been the gold standard, management strategies have evolved with acknowledgement of high surgical morbidity, slow tumor growth rates, and technological advances. Conservative management approaches via observation and newer radiation therapy techniques have become more common. This review seeks to provide an update on contemporary management strategies for HNPGLs and future directions.

## Introduction

**Figure 1:**
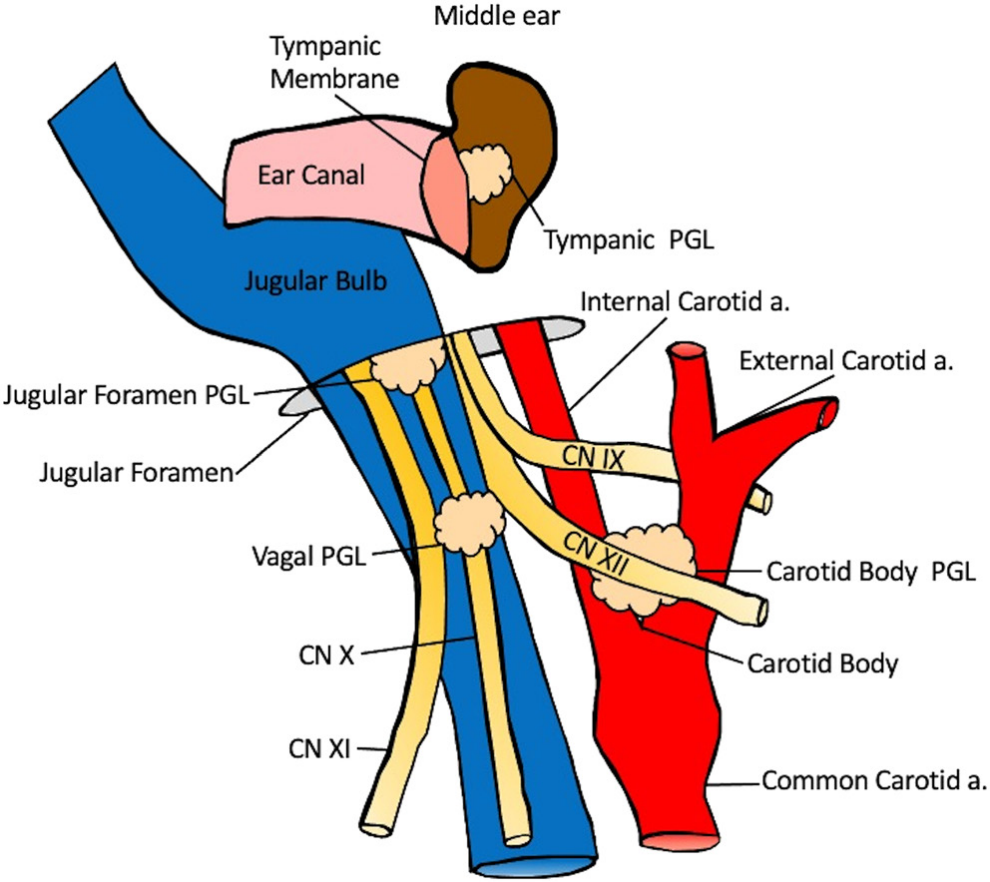
HNPGL subsites. Figure 1 was drawn based on images from Harrison (2009) and Gaillard (2015).

Head and neck paragangliomas (HNPGLs) are rare, slow-growing, vascular tumors arising from cells associated with autonomous ganglia (paraganglial cells) in the head and neck, 95% of which are non-secretory (Fig. 1). Evidence is growing that most paragangliomas (PGLs) are associated with hereditary syndromes in >50% of patients (Horton *et al.* 2022, Richter *et al.* 2022). The most common inherited syndrome involves germline pathogenic variants in genes encoding succinate dehydrogenase (SDH) complex subunits. *SDH* loss promotes cell proliferation, angiogenesis, and tumorigenesis (Gottlieb & Tomlinson 2005). Sporadic tumors are typically solitary, unilateral, and arise between 40 and 70 years of age, while hereditary tumors are more likely multiple and metastatic, and present at younger age (Ikram & Rehman 2022).

PGL subtypes may be categorized by anatomic subsite as either cervical or involving the temporal bone. Cervical PGLs consist predominantly of carotid body tumor paragangliomas (CBTs), glomus vagale paragangliomas (GVs), and, less commonly, cervical sympathetic chain PGLs. CBTs are most common, typically benign, and arise from the carotid body (Offergeld *et al.* 2012). They are often found incidentally on imaging studies or as a painless, palpable, and possibly pulsatile neck mass near the jaw angle. Possible symptoms include hoarseness, dysphagia, and/or autonomic dysfunction due to encroachment of nearby cranial nerves (CNs) IX (glossopharyngeal) and X (vagus). GVs arise along CN X, most commonly from the inferior ganglion. They typically present as an asymptomatic high neck mass but may also present with pulsatile tinnitus and CN IX, X, XI (spinal accessory), and/or XII (hypoglossal) deficits (Offergeld *et al.* 2012 Prasad *et al.* 2019).

Temporal bone PGLs arise from the jugular bulb near the skull base as glomus jugulare paragangliomas (GJs) or in the middle ear cavity as glomus tympanicum paragangliomas (GTs). They most often present with pulsatile tinnitus and/or conductive hearing loss (Carlson *et al.* 2015). Less common symptoms include dysphonia, shoulder weakness/pain, dysarthria, and facial paralysis, all of which should raise greater suspicion for GJs vs GTs (Moore *et al.* 2016). GTs classically present as a red mass behind the tympanic membrane with blanching on pneumatic otoscopy (Sweeney *et al.* 2015).

As mentioned, sympathetic chain PGLs are exceedingly rare but may be considered during diagnostic approach for parapharyngeal masses. Common presenting signs include ipsilateral Horner syndrome (partial ptosis, miosis, and anhidrosis) and oropharyngeal fullness. Sympathetic chain PGLs more commonly secrete catecholamines compared to other PGLs, though all HNPGLs may potentially possess this feature (Seth *et al.* 2014). Other exceedingly rare HNPGL subsites include laryngeal PGLs, arising from the supraglottic or subglottic larynx and presenting with dysphonia or airway obstruction (Myssiorek *et al.* 2004), and parotid gland PGLs (Vora *et al.* 2012).

**Figure 3:**
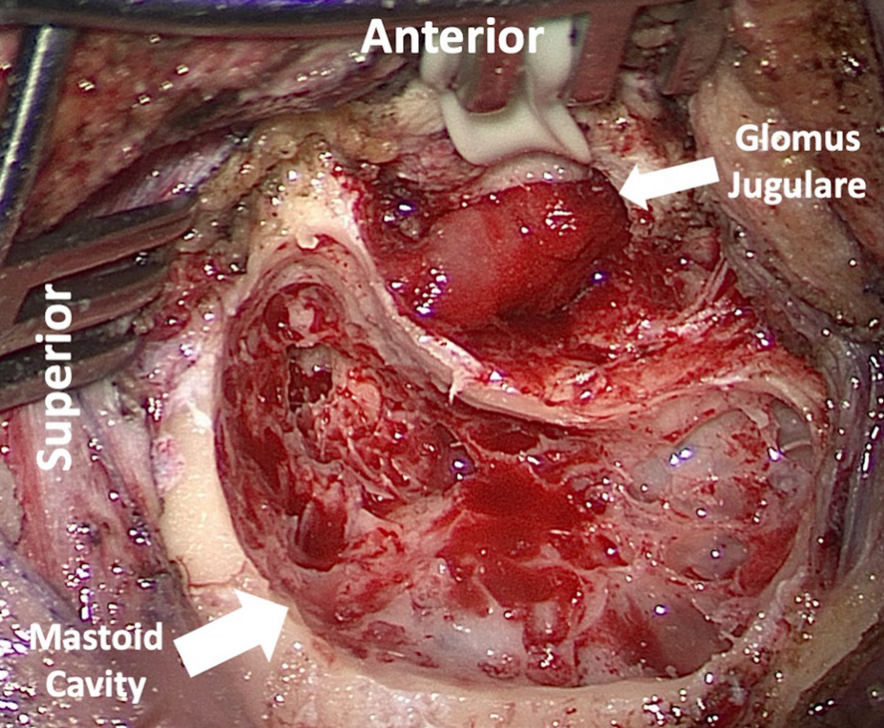
Intraoperative transmastoid approach of glomus jugulare invading up and through the skull base, the middle ear, through the ear canal, and eroding CN VII. The tumor was debulked prior to cable-grafting CN VII and over-closing the ear canal to prevent cholesteatoma.

### Classification

**Figure 2:**
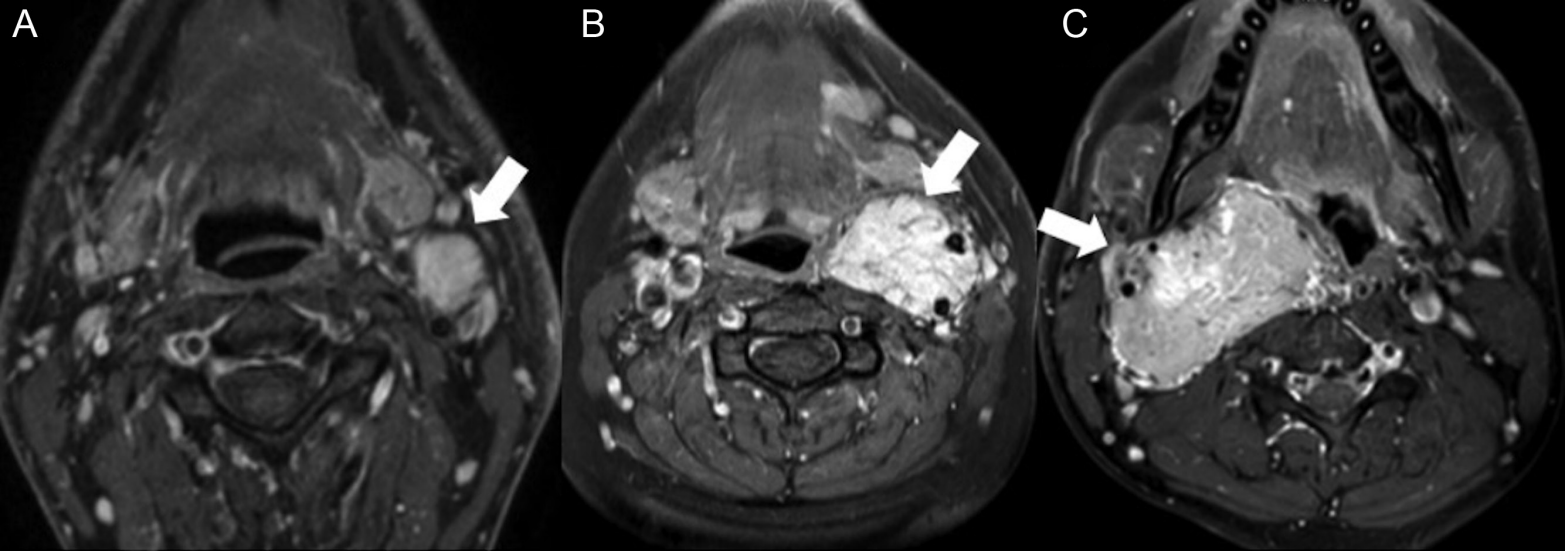
Axial post-contrast MRI findings for Shamblin class I CBT (A), class II CBT (B), and class III CBT (C).

Various classification systems have been proposed for PGLs to improve physician communication and guide operative decision-making. The Shamblin system classified CBTs based on intraoperative anatomical and histopathological findings of tumor size and involvement of the internal carotid artery (ICA) or external carotid artery (ECA). Group 1 tumors are smaller and easily resectable without damage to nearby structures, while Group 2 tumors are larger with greater ICA involvement. Group 3 tumors adhere more to the carotid artery, and its sacrifice is often required during tumor resection (Shamblin *et al.* 1971); (Table 1). However, the Shamblin system is limited by its retrospective nature and inability to guide management, though some use it to predict vascular morbidity (Prasad *et al.* 2019).Arya *et al.* (2008) proposed a modified Shamblin system based on radiographic evidence of carotid artery involvement without consideration of tumor size to determine resectability and guide preoperative decision-making (Fig. 2A-C). No widely accepted classification system exists for GVs (Offergeld *et al.* 2012).

**Table 1:** Shamblin classification for CBTs.

Class	Tumor characteristics
I	Smaller tumors, sparse carotid vessel involvement; completely resectable with minimal morbidity
II	Larger tumors, involvement with or possible infiltration of external and internal carotid arteries; complete resection challenging but possible, likely with greater risk of morbidity
III	Large tumors adherent to and surrounding carotid arteries; complete resection almost always requires vessel sacrifice and reconstruction

The Fisch classification has historically guided surgical approach and resectability of GJs and GTs. Class A tumors are confined to the promontory, class B tumors invade the hypotympanon, class C tumors are subdivided based on the extent of bone erosion into nearby structures, while class D tumors extend intracranially (Table 2).

**Table 2:** Fisch classification for GJs.

Class	Paraganglioma location
A	Tumor arises on promontory
B	Tumor with invasion of hypotympanon; cortical bone over jugular bulb intact
C	Tumor with invasion beyond typanomastoid cavity with destruction of infralabyrinthine and apical temporal bone
C1	Tumor with erosion of carotid foramen
C2	Tumor with destruction of carotid canal
C3	Tumor with invasion of the horizontal part of carotid canal; foramen lacerum intact
C4	Tumor with invasion of foramen lacerum and cavernous sinus
D	Tumor invades intracranially
D1	Tumor with intracranial but extradural extension
D2	Tumor with intracranial and intradural extension

While similar in name and histological structure, HNPGLs differ from thoracoabdominal paragangliomas and pheochromocytomas (PPGLs) by more than location. HNPGLs are typically nonchromaffin, nonfunctional, parasympathetic tumors, while thoracoabdominal PPGLs are often chromaffin and catecholamine-secreting sympathetic tumors (Kimura *et al.* 2021) (Fig. 2).

Several HNPGL treatment options exist. Previously, surgery was considered first-line management; however, high morbidity rates associated with aggressive resection prompted many to favor conservative measures. As knowledge grows regarding how tumor genetics impact prognosis and treatment response, aggressive treatment may become more patient specific. This review focuses on recent reports regarding HNPGL management strategies, including growth rate surveillance, surgical approaches, and radiation therapy.

### Observation/natural history

The general approach to any HNPGL is weighing the risks of surgery, perioperative complications, and irreversible postsurgical sequelae, such as CN deficits, against quality of life (QoL) limitations from the tumor itself. One must also consider metastatic potential – while overall low in HNPGL, it reduces life expectancy, and with progression of disease, it can significantly impact QoL.

CBTs are typically slow growing, with a reported median growth rate of 1.6 mm/year. While CBT growth increases with time, the probability of tumor growth decreases with increased age at diagnosis and higher tumor volume. Observation, therefore, may be indicated for small, benign, asymptomatic tumors, elderly patients, and/or those with surgical comorbidities (Heesterman *et al.* 2017). Nevertheless, for patients ≥60 years old and with minimal comorbidities, surgery remains a viable option at high-volume head and neck cancer centers, with outcomes comparable to patients of younger age (Li *et al.* 2021). The median time to growth is reportedly 1.5 years; surgery may therefore be delayed even in younger patients, if asymptomatic (Heesterman *et al.* 2017). However, observation increases the risk of developing new or worsening symptoms, skull base invasion, carotid artery invasion, and postoperative complications; intervention should occur before such symptoms develop or worsen.

Serial observation is preferred for GVs due to their slow median growth rate of 1.6 mm/year, which is similar to CBTs and greater than temporal PGLs (Jansen *et al.* 2017). Surgery almost always requires CN X sacrifice; resection is thus recommended only after lower CN deficits develop or if the tumor is metastatic or catecholamine secreting, which can be managed with preoperative α-antagonists and β-blockers to avoid hypertensive crisis but cannot be medically managed alone (Shin *et al.* 2012). Observation is often preferred in elderly populations due to slow tumor growth, comorbidities, and greater difficulty with swallowing rehabilitation after high vagal sacrifice (Netterville *et al.* 1998). Observation is also preferred in patients with prior contralateral CN X or XII palsy due to the high risk of ipsilateral CN damage during resection (Shin *et al.* 2012). Surgical resection in bilateral GVs also confers the risk of bilateral lower CN damage; observation is thus preferred until growth is observed, at which point external beam radiotherapy (EBRT) or stereotactic radiosurgery (SRS) may be indicated (Netterville *et al.* 1998).

GJ diagnosis is typically delayed due to its low prevalence, difficult-to-examine location, and slow growth rate (Carlson *et al.* 2015). Once diagnosed, symptoms have often been present for months. GJs are typically benign and have a slow median growth rate of 0.8 mm/year, making observation a preferred management strategy (Carlson *et al.* 2015). Observation may be limited by worsening or new-onset CN deficits (Moore *et al.* 2016). In a case series of 15 GJ patients on observation with a median follow-up of 86.4 months, hearing loss worsened in 38%, pulsatile tinnitus worsened in 0%, CN X function was preserved in 50%, and 69% had normal CN XI and XII function. Under a third of patients had new or worsening lower CN deficits (Carlson *et al.* 2015). Nevertheless, aggressive GJ resection is associated with significant risk of permanent CN IX, X, XI, and XII damage and possibly CN VII paralysis. When indicated, treatment with RT is often recommended to slow tumor growth (van Hulsteijn *et al.* 2013).

While observation is an option, GTs are typically managed surgically. Endoscopic examination and/or serial examinations may be used if patients elect for observation over definitive treatment (Killeen *et al.* 2017). While observation is not widely described in the literature, this strategy likely increases risks of cranial neuropathy and invasive disease.

### Surgical management

When tumor growth or new/worsening symptoms arise, more aggressive measures with surgery may be indicated. Other management options include observation or radiation therapy, while medical oncological therapy is usually reserved for metastatic disease. If a tumor is functional, perioperative α-adrenergic and possibly β-adrenergic blockade might be necessary.

Patients with GJs, GVs, and CBTs may benefit from preoperative embolization (POE) due to the tumors’ highly vascular nature. The nearby ICA and ECA make these tumors easy targets. POE may reduce intraoperative blood loss, operative times, and complications. However, embolization poses a risk for stroke, cranial neuropathies, and vagal or sympathetic chain damage. Although reports of brain embolisms or paradoxical embolisms through PFOs are rare, they are a real risk and embolization should be considered carefully (Griauzde *et al.* 2013).

Reports are conflicting regarding POE efficacy for HNPGLs. Previous studies reported reduced intraoperative blood loss, shorter operative time, and lower complication rates for POE in patients with CBTs and other HNPGLs (Antonitsis *et al.* 2006, Gupta *et al.* 2007, Li *et al.* 2010). However, a recent meta-analysis of 470 patients with CBTs found no significant differences in blood loss, operative time, hospital time, or complications between embolization and non-embolization groups (Abu-Ghanem *et al.* 2016). Failure to completely occlude tumor feeding vessels may increase the risk of revascularization postoperatively; in one report, three such GJ patients had no long-term clinical improvement (Kocur *et al.* 2017). Helal *et al.* (2022) reported that 86% of GJ patients had a >50% tumor blush reduction and zero patients had new or worsened lower CN deficits following POE. Another study reported less perioperative bleeding but a greater CN paresis in GJ patients post-POE (Yildiz *et al.* 2021).Katagiri *et al.* (2019) reported a tumor size reduction of 50% with only transient lower CN deficits in a series of 13 CBT patients. Only one study has compared POE to surgery alone in HNPGLs, in which POE was associated with lower intraoperative blood loss, transfusion requirements, complication rates, and surgical time (Liu *et al.* 2018).

There are various embolization materials, but no randomized trials exist comparing different materials in patients with HNPGLs. In terms of embolization approach, no studies yet have demonstrated superior devascularization with percutaneous, endovascular, or combined approaches (De Marini *et al.* 2021).

Nonetheless, POE appears to be a relatively safe and effective strategy for select HNPGLs. Patients should be selected carefully based on tumor size (>3 cm in diameter), high vascularization, and absence of preexisting lower CN deficits (Colli *et al.* 2021). Alternatively, Jang *et al.* (2018) described a sigmoid sinus tunnel-packing technique in GJ resections that controlled inferior petrous sinus bleeding and improved outcomes with lower costs than POE.

### Surgical goals

#### Cervical PGLs

Gross total resection (GTR) is the gold standard for resectable CBTs in healthy patients due to risks of damage to local structures and risk of metastatic disease if left untreated (Burgess *et al.* 2017). Complete tumor regression with RT or chemotherapy alone is rare (Mendenhall *et al.* 2001). Common complications of CBT resection include ECA ligation, CN XII paralysis, and carotid artery blowout (Fathalla & Elalfy 2020). CN neuropathy risk is highest in tumors >5 cm and/or Shamblin class III tumors due to carotid artery encasement (Arya *et al.* 2008).

Patients with GVs typically present only after symptoms arise, at which point observation usually is no longer indicated. Tumor resection extent then depends on several factors, most importantly age and health status. For elderly but otherwise healthy patients, incomplete resection with lower CN preservation or GTR with vocal cord medialization may be considered (Shin *et al.* 2012). For younger patients, GTR is typically recommended due to their relatively greater tolerance to lower CN deficits, greater response to rehabilitation, and greater risk of recurrence over time from residual tumor (Prasad *et al.* 2019). Unfortunately, CN X is almost always sacrificed during GV resection. In addition to dysphonia and dysphagia, GV resection is associated with ipsilateral pharynx numbness, velopharyngeal insufficiency, CN XII deficits, persistent shoulder pain/weakness, persistent nausea/vomiting, and baroreceptor dysfunction (Moore *et al.* 2016) (Table 3).

**Table 3:** CN involvement by common HNPGL subtypes. The most common findings are bolded.

Tumor type	CN involvement	Possible presenting symptoms	CNs almost always resected/injured
CBP	IX, **X**, XI, XII	**Painless lateral neck mass**. Hoarseness, dysphagia, vertigo, Horner syndrome	Rarely X or XII
VP	VIII, IX, **X**, XI, XII	**Asymptomatic neck mass behind mandible**. Pulsatile tinnitus, hearing loss, dysphagia, shoulder drop, hemiatrophy of tongue	**X** > XII
JFP	VII, **VIII**, X, XI, XII	Facial paralysis, **pulsatile tinnitus, conductive hearing loss,** aural fullness, sensorineural hearing loss and/or dizziness (inner ear invasion), dysphonia/hoarseness, dysphagia/aspiration, shoulder weakness/pain, tongue paralysis	IX > X, XI > XII
TP	**VIII**	**Pulsatile tinnitus, conductive hearing loss**, aural fullness	Rarely VIII

Typically, a transcervical approach is used for cervical PGLs (Fathalla & Elalfy 2020). Transcervical–transparotid approaches may be helpful for tumors extending into the middle parapharyngeal space (PPS), transcervical–transmastoid approaches for those extending into the upper PPS (Fig. 3), and infratemporal fossa approaches for upper PPS tumors extending to the vertical tract of the ICA and jugular bulb (Shin *et al.* 2012, Prasad *et al.* 2019).

#### Temporal PGLs

Traditionally, GJs have been managed with GTR, which yielded high tumor control rates but high morbidity due to their location along the lateral skull base, proximity to lower CNs, local invasiveness, and high vascularity (Gandía-González *et al.* 2014). Postoperatively, patients often experience conductive hearing loss, facial palsy, and lower CN deficits (Bacciu *et al.* 2015). Subtotal resection (STR) strategies have become popularized due to reduced risks of lower CN deficits (Miller *et al.* 2009). STR is often used in conjunction with salvage or adjuvant SRS in instances of residual tumor growth or recurrence (Jansen *et al.* 2018). Several studies have demonstrated lower rates of new-onset lower CN deficits with STR compared to GTR (Manzoor *et al.* 2021). Resection extent in STR varies by disease extent and patient preferences; high-volume tumors extending into cervical and infratemporal fossa locations may require extended STR. Tumor invasion into the middle ear and mastoid or intracranially may prompt limited STR. Limited STR strategies are often performed to limit tinnitus and improve hearing loss by targeted tumor removal. Evidence suggests no relationship between new-onset CN deficits and STR approach (Manzoor *et al.* 2021).

Several surgical approaches for GJs have been described. Historically, translabyrinthine/transcochlear approaches were used but yielded ipsilateral hearing loss (Walker & Babu 2019). Later, infratemporal fossa approach type A became the standard approach for larger tumors due to greater surgical exposure, though this approach still often left patients with a maximal conductive hearing loss (Walker & Babu 2019). For tumors extending intracranially, lateral and posterolateral skull base approaches are helpful while transcranial endoscopic approaches or tympanomastoidectomy may be employed in patients with limited middle ear disease (Manzoor *et al.* 2021). Preoperative imaging should be utilized to determine the best approach for each patient based on tumor size, extent, and patient characteristics.

GTs have traditionally been surgically managed via transcanal and/or postauricular microscope-based approaches, with the latter used predominantly for larger tumors. Evidence has emerged suggesting safe and efficacious GT management via transcanal endoscopic approaches (TEA) for tumors without mastoid bone extension. Fermi *et al.* (2021) reported a 90% GTR rate with few complications and favorable hearing outcomes using TEA. GTR was unachievable in 10% of patients due to tumor extension to the ICA and concern for severe bleeding; however, all patients experienced symptom resolution with no evidence of recurrence during a mean follow-up of 24.3 months. Because morbidity and mortality are limited in surgically managed GTs, radiotherapy (RT) is thought to pose greater risk than benefit (Fermi *et al.* 2021).

### Radiation therapy

#### Goals of radiation

RT has previously been viewed as an adjunct to surgical resection except in select patient populations. For poor surgical candidates, recurrent disease, unresectable locations, and/or palliation, RT can slow tumor growth and reduce the need for surgery (Jackson *et al.* 2001). RT may be reserved for patients with tumor growth on serial imaging due to its serious adverse effects: osteoradionecrosis, secondary malignancy, and vascular ischemic events (Lassen-Ramshad *et al.* 2019). However, in recent years, indications for RT over surgery have grown as newer technology has lowered RT-associated morbidity, while surgical morbidity has gained greater recognition.

#### Conventional external beam radiation therapy vs stereotactic radiosurgery

Different RT techniques have been developed over the years. Previously, conventional EBRT was most commonly used, but has been associated with greater late toxicity rates than newer stereotactic RT (Lassen-Ramshad *et al.* 2019). With technological advances and a movement toward less-invasive HNPGL management, SRS has gained favor, specifically in GJs as it is faster, more convenient, and highly precise and reduces risk of morbidity and lower CN deficits compared to GTR (Lieberson *et al.* 2012, van Hulsteijn *et al.* 2013). As opposed to conventional EBRT where patients receive targeted treatments over weeks, SRS utilizes 3D imaging to deliver single, high-dose, highly precise radiation, limiting collateral damage to nearby healthy tissues. A recent meta-analysis of 1117 HNPGL patients revealed a local control rate of 94.2% after adjuvant or primary SRS (Fatima *et al.* 2021). Pooled estimates of 5-year and 10-year local control was also favorable at 96% and 93.4%, respectively. Clinical neurological symptoms improved in 48.7% of patients, had no change in 39.3%, and worsened in 1.2%. While primary SRS was negatively correlated with lower chances of radiographic local control, one study reported greater correlation with morbidity in surgery than primary RT/SRS and a 78% greater probability of local control than surgery alone (Fatima *et al.* 2021). These studies were limited by relatively short follow-up periods (median 44 months); patients may experience additional morbidity during long-term follow-up. Early SRS adverse effects include transient or permanent lower CN neuropathies, headache, nausea/vomiting, and facial spasm. Importantly, primary SRS does not allow for tissue diagnosis, allowing possible aggressive neoplasms to go undiagnosed (Miller *et al.* 2009).

Other EBRT subtypes have been developed, including intensity modulated radiation therapy (IMRT) and intensity modulated proton therapy (IMPT), which have lower morbidity for at-risk nearby structures. IMPT also spares healthy tissues and further limits adverse effects (Nguyen *et al.* 2021). Compared to IMRT, IMPT adequately covers clinical target volume while offering dosimetric advantages, which theoretically lowers the risk of RT-related morbidity (Nguyen *et al.* 2021). IMRT has been demonstrated as highly efficacious in HNPGLs while limiting morbidity (Anderson *et al.* 2020). Few studies have investigated IMPT efficacy specifically in HNPGLs.

### Special populations

#### Genetic predisposition

There is a strong association between HNPGL and hereditary predisposition to HNPGLs. The most common inherited condition – hereditary PGL syndrome – is caused by a pathogenic variant in one of the genes encoding the subunits of SDH (*SDHA, SDHB, SDHC, SDHD,*and* SDHAF2*). Patients with pathogenic variants in *SDHD* are especially prone to developing HNPGLs, which are often multiple. Overall, a germline pathogenic variant can be found in ~37% of all PGL patients and 55% of those with HNPGLs (Horton *et al.* 2022). Germline *SDHB* pathogenic variants are more often associated with metastatic tumors. However, this does not appear true for HNPGLs. About 8% of HNPGLs are metastatic, with CBT (12%) having the highest rate. Therefore, an *SDHB* germline pathogenic variant should not necessarily influence therapeutic decisions for HNPGLs. However, genetic counseling/testing is recommended for all patients with a PGL as it defines the necessity to screen for other synchronous PGLs, recommends lifelong surveillance, and is the basis for identifying other at-risk family members.

#### Catecholamine-secreting HNPGLs

Catecholamine-secreting HNPGLs are rare with sparse documentation of true incidence rates. This may reflect inadequate biochemical screening following initial HNPGL diagnosis or due to true lack of functional activity by tumors arising from parasympathetic ganglia. We reported a 54.3% biochemical screening rate with a 20.4% positivity rate in new HNPGLs over a 20-year period, possibly due to inadequate initial symptom assessment or patients remaining asymptomatic despite elevated catecholamines (Smith *et al.* 2021). A recent study reports that only 3.7% of all HNPGLs lead to elevated normetanephrine levels. Interestingly, this study also found higher levels of methoxytyramine, the *O*-methylated metabolite of dopamine in benign HNPGLs, which contrasts the prior suggestion of an increase in methoxytyramine and dopamine in metastatic PGLs (Richter *et al.* 2022). Patients with clinically significant catecholamine levels may still present asymptomatically, though symptoms may later arise. Patients should definitively be screened by measuring metanephrines; catecholamine measurement is no longer used. While there is a continued debate about superiority of plasma-free metanephrines vs urinary metanephrines, we prefer to obtain plasma metanephrines. The main reasons are: (i) 24-h urine collection is cumbersome and blood is often drawn during clinic visits anyway and (ii) patients with a PGL have a high pretest probability and thus false-positive results are less likely. Usually, normetanephrine levels must be ≥two- to three-fold elevated to be associated with hypercatecholaminergic symptoms. For borderline positive results, 24-h urine collection or blood draw while supine after 30 min rest can be conducted (Smith *et al.* 2021). Patients with confirmed catecholamine-secreting HNPGLs planning surgical management might need perioperative α-adrenergic and possible subsequent β-adrenergic blockades to prevent intraoperative hypertensive crises, though specific management strategies are often institution dependent, and we recognize the recent discussions around the overall necessity for blockade.

#### Multifocal tumors

It can be difficult to determine whether tumors are truly multifocal or metastatic. In general, if tumors are evident in typical areas in which HNPGLs arise, they are assumed to be multifocal. However, if tumors are seen in organs usually void of paraganglial cells (bone and lymph nodes) they are proven metastatic. Presurgical careful imaging, for example, employing ^68^Ga-DOTATAE PET scans might be helpful in selected patients with higher risk for metastatic disease (e.g. infiltrative growth on cross-sectional imaging and large tumors). The presence of metastasis should influence therapy selection, for example, STR or palliative XRT for symptomatic HNPGL and consideration of systemic therapy for metastatic disease.

Multifocal HNPGL management differs from solitary tumors and poses unique challenges. Patient age should also be considered. Patients >65 years old tend to have greater difficulty compensating for lower CN deficits following surgical resection of HNPGLs – observation with annual imaging may be more appropriate for this population (Moore *et al.* 2016). Tumor location, bilaterality, disease extent, and preoperative lower CN function must also be considered (Szymańska *et al.* 2015). Unilateral multifocal HNPGLs may be approached with a single-step surgical resection (Álvarez-Morujo *et al.* 2016). Bilateral tumors may be managed with a two-stage resection, with the second portion dependent on pre- and postoperative lower CN function to avoid potentially catastrophic complications from bilateral lower CN dysfunction (Szymańska *et al.* 2015, Moore *et al.* 2016). Tumor subtype is also an important consideration in multicentric tumor management. GV resection often requires CN X sacrifice; removal of the larger GV with observation and RT of the smaller tumor are reasonable approaches (Álvarez-Morujo *et al.* 2016). Concurrent bilateral CBT resection should be avoided due to postoperative hypertension resulting from aortic receptor dysregulation, especially when vascular repair or grafts are used; staged resections are thus preferred with >6 months between procedures (Álvarez-Morujo *et al.* 2016). Some surgeons prefer resection of larger tumors first (Velegrakis *et al.* 2001), while others first opt for the smaller tumor (Darouassi *et al.* 2017). For patients with different HNPGL subtypes in varying locations, management may become more complex. We recommend operation on the side with more tumors and/or tumors larger in size. Concomitant HNPGLs and abdominal pheochromocytoma suggest a hereditary syndrome; in addition to the standard genetic testing for *SDHx* mutations recommended for all PGL patients, plasma metanephrines testing should occur prior to surgical intervention (Smith *et al.* 2017).

## Conclusions and future perspectives

HNPGL management approaches have changed dramatically in recent decades. Longitudinal studies have observed great surgical morbidity and conversely demonstrated safety and efficacy of natural observation to preserve lower CN functionality as long as possible. In appropriately selected asymptomatic patients, observation is a reasonable initial approach. While RT has been viewed as an adjunct or alternative treatment for poor surgical candidates, SRS and IMPT may offer local tumor control with lower morbidity compared to surgery and are increasingly viewed as first-line management. Additionally, POE may be less beneficial than previously thought. As knowledge regarding HNPGL genetics grows, management may become more patient specific. Overall, HNPGL management has shifted to more conservative approaches without compromising patient outcomes.

## Declaration of interest

Tobias Else is an associate editor on the editorial board of *Endocrine Oncology*. Tobias Else was not involved in the review or editorial process for this paper, on which he is listed as an author. No other conflicts of interest to declare.

## Funding

None.

## Patient Consent

Written informed consent for the publication of clinical images in Figure 2 was obtained from the patient.

## Author contribution statement

The authors contributed to the conceptualization and design, writing of original draft, review, editing, and approval of final version.
